# Supplementary material for: Depression and its associated factors among health care workers in Saint Paul’s hospital millennium medical college, Ethiopia
Source: PLoS One. 2024 Oct 17;19(10):e0300726. doi: 10.1371/journal.pone.0300726 (PMC11486375; doi:10.1371/journal.pone.0300726)
Supplement: Attachment — Submitted filename: Reviewer comments.docx [file pone.0300726.s003.docx]

Reviewer comments:

- The authors refers to Depressive disorder, but also Depression, Depressive symptoms, Depressed symptoms, Major depressive disorder etc… interchangeably, which can be confusing to the reader. These phenomena are not the same thing, and it is advisable to use one term consistently throughout the abstract and main text. In this case, either Depression or Depressive symptoms.
- In Paragraph 3 of the background - it is important to clarify that those studies were also done among HCW. Only the first sentence mentions HCW.
- The last paragraph of the background is too repetitive. The aim of study only needs to be stated once.
- In the sample size section, it is not clear how the authors came up with this method of sample size calculation. If this is a pre-existing method then a citation needs to be added, otherwise if this is an entirely new method of size calculation that also needs to be stated.
- In the Statistic Analysis, name should be Microsoft Excel, and mention the version used
- For the results, the authors started very well using past tense (were, was) but then later on switched to present tense (are, is, has). When writing results and discussion, the standard rule is to use past tense because we are mentioning what was found at the time of data collection, not now.
- Decide whether you want to use the name in full, or abbreviation SPHMMC, but not both
- What do the authors think about HCW responding to questions about suicide attempts, and chronic health issues? Could this be limitation? How did they address it?
